# Exploring the Role of Sustainable Development Goals in Enhancing Courage, Proactive Career Behaviors, and Life Satisfaction

**DOI:** 10.3390/bs14090843

**Published:** 2024-09-19

**Authors:** Anna Parola, Cristiano Felaco

**Affiliations:** 1 Department of Humanities, University of Naples Federico II, 80133 Naples, Italy; 2 Department of Social Sciences, University of Naples Federico II, 80138 Naples, Italy; cristiano.felaco@unina.it

**Keywords:** SDGs, proactive behaviors, life satisfaction, well-being, future career, young adults

## Abstract

Understanding the current challenges addressed in the goals of the 2030 United Nations Agenda can influence career choices, encouraging individuals to pursue careers that contribute positively to addressing them. This study examines the association between the propensity to consider the Sustainable Development Goals (SDGs) in relation to future educational and career paths, courage, proactive career behaviors and life satisfaction, and the mediating role of courage and proactive career behaviors on the association between the propensity to consider the SDGs in relation to future educational and career paths and life satisfaction. The study sample consisted of 314 Italian university students. The serial multiple mediation model was used to examine the direct, indirect, and total effects. The results showed that the propensity to consider SDGs in relation to future educational and career paths, through courage and proactive career behaviors, has a positive impact on life satisfaction. The findings of this study have led to several actionable policy recommendations. These advocate for the integration of activities related to modules on the SDGs into their curricula. In addition, practical implications for career guidance interventions are proposed to consider the role of the SDGs in future career planning.

## 1. Introduction

Against the backdrop of growing global concerns about sustainable development, it is important for individuals to align their career paths with broader societal goals. Imagining one’s future career plans today necessarily means taking into account the unstable context in which young adults have to navigate. Indeed, the context and its challenges and threats must be considered, as they determine the outcomes of young adults’ transitions into the world of work [1,2]. Factors such as technological advances, economic downturns, and environmental changes are often cited as major threats, leading to increased labor market uncertainty and competition, job insecurity, and fragmented career paths [3]. In addition, the consequences of the pandemic should not be underestimated. The COVID-19 pandemic has exacerbated a global sense of uncertainty [4]. On the one hand, the literature argues that young adults are and will continue to be the group most exposed to economic hardship as a result of the pandemic [5]. The long-term effects of the pandemic will threaten the transition from school to work. On the other hand, it is also worth considering how the pandemic has changed the world of professions and the need to know how to work under emergency conditions. Several studies have shown that professions themselves and the skills required are different: for example, the climate in which health professionals have been working [6], the massive introduction of online learning for teachers and students [7], and smart working [8]. These changes have led to an increasing need to improve professional knowledge and skills in university courses and their beliefs about the value and meaning of their future profession [6].

Effective career guidance has been identified as key to shaping societies and the individuals who live in them [9,10]. In recent years, career development theories and career guidance practices have highlighted the need to equip adolescents and young people for their future careers in order to ensure global sustainable development [11]. The concept of sustainable development is not new. Since the 1980s, this theme has been concerned with balancing the social, economic, and environmental aspects of development [12]. In the Brundtland report of 1987, entitled “Our Common Future”, sustainable development is delineated as advancement that satisfies the present generation’s necessities while safeguarding the potential of forthcoming generations to meet their own needs [13]. In 2000, these premises led to the establishment of eight measurable Millennium Development Goals (MDGs), with targets to be achieved by 2015 [14] aimed at tackling poverty, hunger, disease, and inequality. The MDGs are the precursors of the current Sustainable Development Goals (SDGs) outlined in the 2030 Agenda for Sustainable Development. The 2030 Agenda, which has been ratified globally by the United Nations, seeks to advance a plan for sustainable development on a world scale, founded on three pillars—people, planet, and prosperity—by fostering collaboration between different sectors and nations. The SDGs present a set of priority actions consisting of 17 goals, 169 targets, and 232 indicators that address a spectrum of socio-economic and environment-related development issues, such as reducing poverty, improving the quality of education, promoting gender equity, reducing inequalities, supporting decent work and economic growth, addressing climate change challenges, and ensuring peace and justice. These goals are interlinked, as progress in one field often depends on progress in others. Achieving the SDGs will therefore require cooperation among various entities, including governments, corporations, civil society, and citizens, at local, national, and global levels. The SDGs are integral to shaping the future sustainability of society by providing a roadmap for addressing global challenges and promoting inclusive, equitable, and sustainable development around the world.

The future’s global challenges and the well-being of people are the leitmotifs of current career guidance interventions, which aim to help individuals shape their future in a sustainable and inclusive society. In recent years, several books have highlighted the importance of the strong link between career education, career guidance and the SDGs [15,16], providing insights into how novel forms of education and career interventions can build a more inclusive and sustainable future society for all [15]. With this in mind, the career guidance interventions should take into account the aspirations, desires, attitudes and skills of the clients and the extent to which they can contribute to a sustainable society for all. According to Di Maggio et al. [17], young people should be encouraged to be more oriented towards future career activities that allow them to achieve their well-being and contribute to the realization of inclusive and sustainable environments. Nevertheless, there are still few studies that attempt to examine the role that keeping the goals of the 2030 Agenda in mind can play in building one’s career, in terms of adopting proactive career behaviors with courage and achieving psychological well-being. In light of this, the present study aims to fill this gap by exploring the role of the propensity to consider the goals of the 2030 Agenda in one’s future choices as a guiding force for enhancing courage, proactive career behavior, and well-being, providing useful information for planning career interventions in educational contexts.

## 2. Theoretical Framework

The framework of Career Construction Theory (CCT; [18]) emphasizes the challenges that adolescents and young adults face today in choosing their future career plans. Indeed, designing future careers means taking into account one’s desires and aspirations for the future but also coming to terms with the social, economic, and environmental changes that shape the environment where career transitions occur. Based on the current work of the world scenario in which the transition from stability to unpredictability has occurred, adolescents and young people should rely on their resources, called career adaptability, to face unpredictable situations by making changes. In CCT, career adaptability includes the four C’s: concern about desirable futures, control in managing the relationships with the personal environment, curiosity in exploring and improving the environment, and confidence in one’s ability to overcome challenges. In this way, individuals would be able to cope with the challenges that may arise in the pursuit of their goals and have the opportunity to influence the environment through personal actions [19].

Especially in the last decade, career guidance models have become increasingly concerned with how an individual’s choice of their future impacts everyone and the future planet. The Life Design paradigm, which arises in CCT as a career intervention, suggests the importance of each individual to design their own life [20]. Alongside this, the lifelong perspective also provides an opportunity to reflect on how any life project of the individual must necessarily also consider the collective human experience, the essence of humanity, and the state of the world. In a recent article, Guichard [11] tries to answer the question, “How could interventions for life- and career-construction contribute to a development that would be ecologically sustainable, socially just and based on decent work activities?” (p. 583). Guichard [11] poses the reflection on “toward what futures” the career guidance practices are orienting young people by emphasizing the urgency of keeping in mind that the guidance should drive toward three dimensions: living on (“How can I ensure my survival?”), self-realization (“What working life could give me a good life?”), and acting reasonably (“What can I do to contribute to a collective life that is good and just?”). Interventions (“action life design” [11]) should include (a) learning about the role of work, forms of organizing, and exchanging in the construction of the subjectivity of each individual and world, and (b) reflecting on how each person can work to achieve the SDGs [21]. Thus, current career construction theories seek to consider and weigh the 2030 Agenda’s goals.

The literature investigating how effectively awareness of the influence of the SDGs on the construction of future career plans can influence career outcomes is still limited. While several articles have examined the importance of working with the SDGs for successful career guidance (e.g., [17,21,22,23,24,25,26]), few empirical studies have quantitatively investigated such outcomes. In accordance with CCT, a sequence of adaptation is outlined that begins with adaptive readiness for change, then with adaptive responses, and finally with adaptive outcomes [18]. As state by Savickas and Porfeli, “People are more or less prepared to change, differ in their resources to manage change, demonstrate more or less change when change is needed, and as a result become more or less integrated into life roles over time” ([19], pp. 661–662]). This study sought to understand, within this paradigm, whether increased awareness of the SDGs could lead adaptive responses, and finally adaptive outcome. Adapting responses are actual behaviors that help individuals to meet changing conditions, while adaptation results are the successful outcomes of adapting [19]. As adaptive responses, this study considers courage and proactive career behaviors. As will be detailed below, courage is an adaptive behavior to cope with career development, which, in turn, can set proactive career behavior in motion. As an end result, and thus an adaptive outcome, this study considers life satisfaction.

### 2.1. Proactive Career Behaviors

Proactive career behaviors indicate individuals’ engagement in various activities aimed at proactively developing their careers [27]. These behaviors are future- and change-oriented [28]. Several studies have confirmed that proactive career behavior leads to the achievement of desired career outcome success [29]. Exploring whether awareness of the SDGs is a driver of proactive career behaviors is an important contribution for both practitioners in the field of career counseling and those involved in career guidance programs for young graduates in the labor market.

Taking a proactive approach to developing your career is crucial to succeeding in the current environment [29,30]. Hirshi [31] demonstrated how high levels of career proactive behavior support an adaptive college-to-work transition. Personal and environmental factors may influence career proactive behaviors. While the existing literature has mainly paid attention to the dispositional factors of proactive career behaviors, such as personality [32], self-efficacy [27], and hope [33], few studies have explored the role of environmental factors, particularly perceived career barriers [34]. However, environmental factors may be critical in determining the adaptive responses [34,35]. According to CCT [18], career proactive behaviors are an individual’s adapting responses. Adapting responses are concrete behaviors aiding people in navigating shifting circumstances, which can lead, in turn, to positive adaptation results [19,35,36,37]. Specifically, individuals tend to take proactive measures to manage their career development, like career planning, requesting guidance, and learning new competences, when they proactively undertake career behaviors [38].

### 2.2. Courage

Alongside proactive career behaviors, courage serves as a powerful protective factor in negotiating career transitions and shaping future trajectories [31]. Courage has traditionally been studied as a trait and as a process [39]. The concept of courage as a trait requires objective, external standards to determine the objective risk and to define an action as courageous. The concept of courage as a process requires the subjective evaluation of the action from the point of view of the person experiencing it. 

In the CCT paradigm, courage functions as an “adaptive behavior to cope with career development tasks and changing work” ([40], p. 459). Several studies have conceptualized courage as the willingness to engage in behaviors despite experiencing fear [41,42]. This perspective allows courage to be seen as a vital response by individuals to cope with their context. Parola and Marcionetti [43] suggested that courage can manifest itself in actions that foster a sense of protective engagement with one’s professional endeavors, thereby helping individuals to maintain their well-being. Studies have shown the relationship between courage and various career resources, such as career adaptability [43], resilience [44], optimism, and hope [40], and career outcomes, such as job performance [45] and future orientation [40,46], satisfaction, and well-being [47,48,49]. Interventions that help to build or strengthen courage have the potential to improve the mental health of adolescents and young people in their career transitions [43]. In this way, courage can be seen as a strong protective factor in school-to-work transitions. 

### 2.3. Life Satisfaction

Investing in sustainable development is critical to addressing global challenges and ensuring well-being and satisfaction [50]. Costanza [51] highlights the importance of establishing a collective vision of a sustainable and desirable society as an essential endeavor. Glasser [52] defines the importance of the Sustainable Well-being Revolution as an effort to prioritize the well-being of all individuals, which must be a practical goal. Awareness of the influence of the SDGs could contribute to well-being by instilling a sense of hope for the future and motivating individuals to create a sustainable and inclusive world, which would have a positive impact on their well-being.

Life satisfaction is commonly used as an indicator of well-being [53]. It refers to how individuals evaluate the overall quality of their lives and represents the cognitive aspect of subjective well-being [54]. Unlike emotional components (positive and negative affect), life satisfaction tends to be less sensitive to mood fluctuations and slower to respond to changes in life circumstances [55]. Achieving these SDGs brings about social, economic, and environmental transformations, which serve as prerequisites for sustainable development [56]. As a result, such changes may influence life satisfaction [57,58]. Kubiszewski et al. [59] found that economic, social, and environmental indicators of the SDGs influence life satisfaction. Ortega-Gil et al. [60] showed a positive relationship between circular economy, environmental taxes, and spending on environmental protection and life satisfaction. Salonen [61] also highlighted the importance of education in promoting sustainability and life satisfaction. Finally, in a recent study, da Silva et al. [62] found that compliance with the SDGs influences life satisfaction.

### 2.4. Links between the SDGs, Proactive Career Behaviors, Courage, and Life Satisfaction 

The key question is, what is the relationship (if any) between SDGs awareness, adaptive responses (courage and proactive career behaviors), and adaptive outcomes (life satisfaction)? Following CCT [18], an individual who is aware of the characteristics of the SDGs, for example, decent work, respecting the environment, and promoting gender equality (to mention a few of the goals of the Agenda), spurs people to take practical actions that can be classified as courageous. Courage, in turn, stimulates people to protect what is important to them, and having, for example, a decent job, as well as respecting the environment and promoting gender equality, can be important elements that drive action. Thus, understanding whether awareness of the SDGs triggers courage, which in turn drives proactive behaviors and leads to perceived satisfaction, allows us to understand whether and how important it is to think about interventions outside and within educational settings that promote reflection on current challenges and shared sustainable goals.

As mentioned above, previous research has linked awareness of the SDGs to life satisfaction [62], while no studies have examined the relationship between the SDGs and courage and proactive career behaviors. Studies within the CCT framework have primarily focused on the association between the propensity to believe that the SDGs will influence future education and careers with adaptive resources, such as adaptability [50]. The relationship between courage and well-being has been established in several studies [40,43,63]. In addition, Parola et al. [47] demonstrated the mediating role of courage between career calling and well-being outcomes, i.e., life satisfaction and flourishing. Lodi and colleagues [48] demonstrated the mediating role of courage between optimism and academic satisfaction. Magnano et al. [49] found this mediating role between employability and well-being. Finally, the link between proactive behaviors and life satisfaction has been found in previous studies [27]. In addition, proactive behaviors have been associated with several adaptive responses, such as optimism and hope [27,33]. However, the relationship between courage and proactive behaviors remains unexplored. 

### 2.5. Study Aims and Hypothesis 

While the importance of considering the SDGs in career development is widely acknowledged, quantitative studies exploring this relationship are scarce, especially with regard to the study of their relationship with adaptive responses. Indeed, while the relationship between the SDGs and life satisfaction has been confirmed by previous studies [62], the relationship between the SDGs and specific important adaptive responses—in this case, courage and career proactive behaviors—has never been explored. The present study aims to fill this gap by investigating the relationship between the propensity to believe that the SDGs will affect one’s future career and adaptive responses, i.e., courage and career proactive behaviors, as well as life satisfaction, as an indicator of well-being. Additionally, this study examines the mediating role of these adaptive responses between the propensity to consider that the SDGs influence future career and life satisfaction. Based on the outlined literature and drawing on the theoretical framework of CCT, an increased awareness of the role of the SDGs in future career plans can increase courage in making career choices, promote proactive career behaviors, and, in turn, lead to life satisfaction. Therefore, courage and proactive career behaviors can act as sequential mediators, first courage and then behaviors, in the relationship between the SDGs and life satisfaction. 

Explicit hypotheses about each relationship (path) between study variables were formulated:

**H1a:**  *The SDGs (X) have a direct positive association with life satisfaction (Y).*

**H1b:**  *The SDGs (X) have a positive association with courage (M1) and proactive career behaviors (M2).*

**H1c:**  *Courage (M1) has a direct positive association with life satisfaction (Y).*

**H1d:**  *Proactive career behaviors (M2) have a direct positive association with life satisfaction (Y).*

**H1e:**  *Courage (M1) has a direct positive association with proactive career behaviors (M2).*

**H2a:**  *Courage (M1) mediates the relationship between SDGs (X) and life satisfaction (Y).*

**H2b:**  *Proactive career behaviors (M2) mediate the relationship between SDGs (X) and life satisfaction (Y).*

**H3:**  *The relationship between the SDGs (X) and life satisfaction (Y) is sequentially mediated by courage (M1) and proactive career behaviors (M2).*

## 3. Materials and Methods

### 3.1. Participants and Procedure of Data Collection

A total of 314 young adults (57.1% females and 41.9% males) took part in this study. The participants were between 18 and 24 years old (M = 21.2; SD = 0.46) and were pursuing a bachelor’s degree across southern Italian universities. A total of 40% of them were equally distributed in science fields (computer science, math, physics), and 60% in humanities ones (sociology, political science, psychology). 

The questionnaire was administrated at the beginning of the university lesson and completed in the presence of two researchers involved in the study. Participants were provided with a QR code to fill out the questionnaire online. Prior to data collection, participants received a comprehensive explanation of the study’s aims and were encouraged to join, while ensuring their anonymity and the freedom to withdraw at any time.

### 3.2. Measures

#### 3.2.1. Socio-Demographic Information

At the beginning of the questionnaire, participants were invited to report socio-demographic information, respecting anonymity and privacy. Specifically, students were asked to indicate their age, gender, and degree (curriculum) being pursued.

#### 3.2.2. The Propensity to Consider the SDGs in Relation to Future Education and Career Paths

To assess the propensity to consider the SDGs tied to future educational and career paths, the measure “Goals for the Future Design of the 2030 Agenda” (GFD; [50]) was used. The questionnaire consists of 17 items, one for each goal of the 2030 Agenda. Examples of items are, “How much could the topic of access to quality education for everybody influence your future education and career design?” and “How much could the topic of promoting decent work influence your future education and career design?” Participants are invited to report the influence of each goal on their educational and career design on a 5-point Likert scale ranging from 1 (=almost not at all) to 5 (=very much). The scale score was created as the mean of the questionnaire responses over the set of individual items. A higher score indicates a greater propensity to consider the SDGs for future career choices. In this study’s sample, the Cronbach’s alpha was 0.87.

#### 3.2.3. Courage

To assess courage, the Italian version of the Courage Measure [40,42] was used. This measure is often used to measure individuals’ propensity to behave courageously in overcoming career-related challenges. The questionnaire consists of 6 items (e.g., “If there is an important reason to face something that scares me, I will face it”). Participants are invited to respond on a 7-point Likert scale ranging from 1 (=never) to 7 (=always). The scale score was created as the mean of the questionnaire responses over the set of individual items. A higher score indicates greater courage. In this study’s sample, the Cronbach’s alpha was 0.91.

#### 3.2.4. Proactive Career Behaviors

To assess proactive career behaviors, the Career Engagement Scale [27] was adopted. The questionnaire is composed of 9 items (e.g., “Undertook things to achieve your career goals,” “Cared for the development of your career”). Participants are requested to indicate the level of engagement in this task over the previous six months on a 5-point Likert scale ranging from 1 (=almost never) to 5 (=very often). The scale score was created as the mean of the questionnaire responses over the set of individual items. A higher score indicates greater proactive career behaviors. In this study’s sample, the Cronbach’s alpha was 0.88.

#### 3.2.5. Life Satisfaction

To assess life satisfaction, the Satisfaction with Life Scale (SWLS, [64,65]) was used. The measure consists of 5 items (e.g., “In most ways, my life is close to my ideal”). Participants are asked to respond on a 7-point Likert scale ranging from 1 (=strongly disagree) to 7 (=strongly agree). The scale score was created as the mean of the questionnaire responses over the set of individual items. A higher score indicates a greater level of life satisfaction. In this study’s sample, the Cronbach’s alpha was 0.89.

### 3.3. Statistical Analysis

In the first step, preliminary analyses were conducted. Specifically, means and standard deviations were determined. Skewness and kurtosis were determined to test the normality of the distribution. 

Correlation analyses between gender, university curriculum, the SGDs, courage, proactive career behaviors, and life satisfaction were carried out to assess the relationships between the study dimensions. The following benchmarks were used: *r* < 0.10, trivial; *r* from 0.10 to 0.30, small; *r* from 0.30 to 0.50, moderate; and *r* > 0.50, large [66].

**Figure 1:**
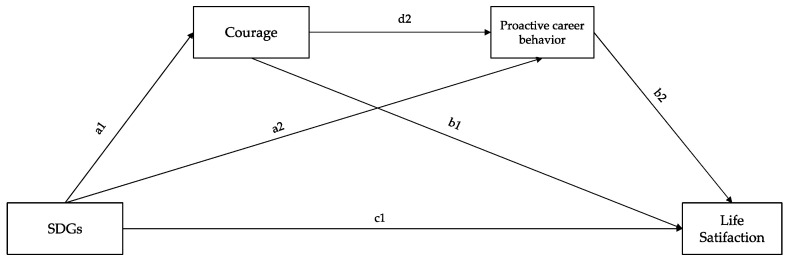
Hypothesized model. Note: “SDGs” indicates the propensity to consider the SDGs in relation to future education and career paths.

In the second step, starting from the hypothesized model (Figure 1), a serial mediation model with observed variables was run through Model 6 of Process Macro [67]. A two-step approach [67,68] was used. In Step 1, the predictor-only model was specified: The SDGs (X) were regressed on life satisfaction (Y). In Step 2, the full sequential mediation model was specified: The SDGs (X) were regressed on life satisfaction (Y) via courage (M1) and proactive career behaviors (M2). Gender and university curriculum were included as covariates in the model.

To assess the indirect effects, a percentile bootstrap estimation approach (95% confidence intervals) with 5000 samples was adopted. The model used standardized variables. The reported regression coefficients were unstandardized in the text (β) and standardized in the figure (b).

## 4. Results

Preliminary analyses of the skewness and kurtosis revealed the normality of the distribution. Correlation analyses are displayed in Table 1.

The results revealed moderate to large correlations between the study variables. Specifically, a large correlation emerged between life satisfaction and proactive career behaviors (*r* = 0.573) and a moderate correlation between courage (*r* = 0.397) and the propensity to consider the SDGs in relation to future education and career paths (*r* = 0.463). A large association also emerged between proactive career behaviors and the propensity to consider the SDGs in relation to future education and career paths (*r* = 0.566) and a moderate correlation with courage (*r* = 0.374). Finally, a moderate correlation between courage and the propensity to consider the SDGs in relation to future education and career paths emerged (*r* = 0.314). 

With regard to socio-demographic information, no significant relationship emerged for gender and study variables, except for a weak relationship with courage (*r* = 0.112). There were no correlations between university curriculum and study variables.

Mediation analyses were set up to detect the predictive role of the propensity to consider the SDGs in relation to future education and career paths and life satisfaction. A serial model was then tested to capture the mediating role of courage and proactive career behaviors between the propensity to consider the SDGs in relation to future education and career paths and life satisfaction. Gender and curriculum were inserted as covariates in the model. 

The serial multiple mediator analysis showed the direct effect of the propensity to consider the SDGs in relation to future education and career paths on life satisfaction (β = 0.413, SE = 0.132, 95% C.I. [0.153, 0.673]), courage on life satisfaction (β = 0.273, SE = 0.068, 95% C.I. [0.139, 0.407]), and proactive career behaviors on life satisfaction (β = 1.278, SE = 0.177, 95% C.I. [0.931, 1.626]). Furthermore, the relationship between the propensity to consider the SDGs in relation to future education and career paths and courage (β = 0.548, SE = 0.093, 95% C.I. [0.365, 0.730]) and proactive career behaviors (β = 0.383, SE = 0.036, 95% C.I. [0.312, 0.455]) were shown. Finally, the results showed a significant relationship between courage and proactive career behaviors (β = 0.092, SE = 0.021, 95% C.I. [0.050, 0.134]). 

With regard to the effect of covariates, no significant effect was found for the curriculum variable. For gender, only an effect of the variable on courage emerged, in favor of female gender (β = 0.232, SE = 0.105, 95% C.I. [0.026, 0.439]).

**Figure 2:**
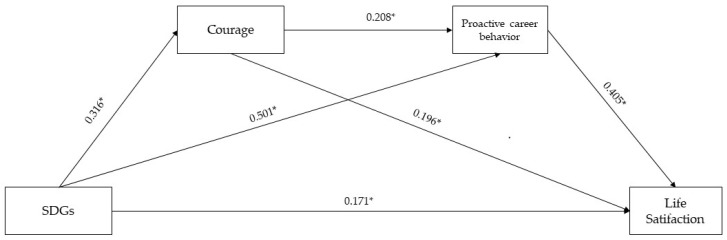
Serial multiple mediation analyses (standardized beta coefficient). Note: “SDGs” indicates the propensity to consider the SDGs in relation to future education and career paths; * *p* < 0.001.

The total effect of the propensity to consider the SDGs in relation to future education and career paths on life satisfaction was significant (β = 1.117, SE = 0.121; 95% CI [0.878, 1.356]). The indirect effect of model 1 (single mediation effect through courage) was significant (β = 0.149, SE = 0.051; 95% C.I. [0.066, 0.258]). The indirect effect of model 2 (single mediation effect through proactive career behaviors) was significant (β = 0.490, SE = 0.121; 95% C.I. [0.275, 0.750]). Finally, the indirect effect of model 3 (the serial mediation effect through courage and proactive career behaviors) was also significant (β = 0.064, SE = 0.026; 95% C.I. [0.019, 0.121]). The total variance explained (*R*^2^) was 0.389. The results of these analyses are presented in Figure 2.

## 5. Discussions

### 5.1. The Relationship between the SDGs, Courage, Proactive Career Behaviors, and Life Satisfaction

Correlational analyses revealed a significant positive relationship between the propensity to consider the SDGs in relation to future educational and career paths and life satisfaction, courage, and proactive career behaviors. These findings emphasize the relevance of awareness of the SDGs in constructing educational and career futures, which increases courage in enacting career behaviors and well-being. Similarly, a significant positive correlation was observed between courage and proactive behaviors, indicating that increased courage in decision-making enhances the likelihood of engaging in actions to pursue one’s goals. Finally, courage and proactive behaviors are also significantly and positively associated with higher perceived life satisfaction.

### 5.2. The Predictive Role of the SDGs on Life Satisfaction

The serial mediation model was conducted to test our theoretical model. Specifically, the model tested the predictive role of the propensity to consider the SDGs in relation to future educational and career paths on life satisfaction and the mediating role of courage and proactive behaviors between the propensity to consider the SDGs in relation to future educational and career paths and life satisfaction. The model confirmed our hypotheses. Indeed, the results detected the predictor role of the propensity to consider the SDGs in relation to future educational and career paths on life satisfaction and the mediating role of courage and proactive behaviors between the propensity to consider the SDGs in relation to future educational and career paths and life satisfaction. Regarding the first hypothesis (H1a), the results are consistent with previous literature that found a relationship between SDG compliance and life satisfaction [62]. Awareness of the SDGs can provide individuals with a sense of purpose and meaning, contributing to their perceived competence to make meaningful contributions to global challenges. By understanding the mission outlined in the SDGs, young adults may experience a greater sense of purpose. Indeed, awareness of the interconnectedness of global issues and one’s role in addressing them through their educational choices and future career paths can imbue life with greater meaning and significance, contributing to life satisfaction. This evidence ties in with studies that find how individuals choose their careers ties into the well-being of others and society. Several qualitative studies have shown how the well-being of future societies plays a crucial role in young adults’ career choices [69], highlighting the significance that work is perceived to have as a personal and social good [70]. Satisfaction with life serves as a significant resource for individuals in planning their future [71].

### 5.3. The Predictive Role of the SDGs on Courage and Proactive Career Behaviors

Interestingly, and in line with our hypothesis (H1b), our findings suggest that the propensity to consider the SDGs in education and career choices leads to increased courage. In this model, courage is defined as the extent to which individuals can overcome fear to make decisions. Similarly, the link confirmed between the propensity to consider global challenges in educational career choice and proactive career behaviors is novel in the literature. Proactive career behaviors in CCT represents the disposition to effect environmental change by taking initiative in a broad range of situations. In this sense, this propensity of the individual is predicted by awareness of the 2030 goals, thus posing as an important indicator of the individual’s adaptation to the environment. 

Consistent with our hypothesis and current literature on the CCT paradigm [27,40,43,63], the results also suggested the predicting role of courage on life satisfaction (H1c) and between proactive career behavior and life satisfaction (H1d). The relationship between courage and proactive career behaviors is also confirmed (H1e), suggesting that young adults who experience courage in engaging in activities are associated with being proactive.

### 5.4. The Mediating Role of Courage and Proactive Career Behaviors

Furthermore, our model shows that courage (H2a) and proactive career behaviors (H2b) act as mediators in the relationship between the SDGs and life satisfaction. In addition, in line with our serial model hypothesis (H3), courage and proactive career behaviors sequentially mediate the relationship between the propensity to consider the SDGs in relation to future educational and career paths and life satisfaction. The results indicated that young adults who are inclined to consider the influence of the SDGs on future careers demonstrate greater courage in shaping the future, resulting in higher levels of career engagement and, consequently, greater perceptions of life satisfaction. 

These findings underscore the importance of integrating reflection and awareness of the 2030 Agenda goals into career development. As acknowledged in the literature and noted above, young people navigate a complex socio-economic landscape characterized by significant challenges, which can result in fragmented career paths [3]. Consequently, career choices have become increasingly daunting for the younger generation, often leading to pronounced career indecision [72] and feelings of insecurity about their future [73]. Furthermore, these challenges have been linked to inactivity among young adults [74,75]. Our findings suggest that fostering awareness of the SDGs may act as a catalyst for career engagement, leading to adaptive career responses and results. Raising awareness among adolescents and young adults about the challenges and goals of the 2030 Agenda means working on resources for career adaptability. In line with this, there is a consensus in the literature that a higher tendency to consider the SDGs important for future career planning is associated with higher levels of career adaptability [50]. Awareness of future challenges and the goals of the 2030 Agenda can motivate people to develop careers that are more aligned with the future needs of the planet and society, making them not only better prepared to cope with change but also to make a positive contribution to the future environment.

### 5.5. Insights for Career Construction

Being aware that the SDGs should play a role in choosing future careers means being more aware that one’s actions can positively impact the future. Knowing how one can contribute to global goals such as poverty reduction, health promotion, and equality can increase personal fulfillment and professional satisfaction. For example, if young people are aware of ways to safeguard the planet, they will consider pursuing future ecologically sustainable jobs. Similarly, they will be able to make choices leading them towards decent jobs that uphold equal rights in the world of work. 

Furthermore, feeling like a part of a global movement for positive future change can enhance engagement and motivation in educational pursuits. Individuals are more likely to be motivated to pursue their careers when they perceive their work as contributing to a meaningful goal [76]. In essence, alignment with the SDGs can inspire individuals to explore career paths and opportunities that contribute to sustainable development, encouraging proactive career choices and a proactive approach to seeking relevant training or educational pathways. Individuals can actively seek opportunities to develop skills and competencies that are in demand in emerging fields related to sustainability, enabling them to navigate changing market demands and capitalize on career opportunities. The entire model shown here takes into account the possible key factors in adaptation, i.e., courage and proactivity. Thus, awareness of the challenges of the environment and its role in future career choices would, according to CCT [18], lead to the enactment of adaptive behaviors that are essential for individual–environmental adaptation in the direction of well-being.

## 6. Practical Implications

### 6.1. Implication for Career Guidance 

This study has important practical implications for career education and guidance. Educational systems, such as secondary schools, should include modules on the SDGs in their curricula. These modules should not only cover what the Sustainable Development Goals are but also facilitate discussions on how they can contribute to building better societies for all. The educational system is recognized as an ideal environment to promote activities that support students in their career exploration, taking into account the role of the SDGs in empowering individuals and encouraging them to engage as active agents of social change [26]. Additionally, career practitioners should consider the impact of the SDGs on future careers, which can encourage individuals to engage in strategic career planning. Through the lens of CCT [18] and the Life Design paradigm [20], a clearer connection emerges between the SDGs and the opportunity and need to address them in career construction. Indeed, Life Design is conceived as a prevention device and serves to promote the acquisition of awareness of one’s own resources and capabilities to create one’s own path in the face of the current challenges. Life Design interventions are designed on the basis of constructionist approaches and thus take into account current and future societal challenges, inducing new self-evaluations and perspectives among young people [77,78,79]. As Nota and colleagues suggest, “Career counseling should encourage people to consider the external reality and anticipate future trends, while acknowledging that these factors cannot be solely interpreted or manipulated based on one’s own interests, passions, and human capital” ([16], p. 47). Advocating for the positive role of considering global challenges in adaptive career responses and well-being does not imply narrowly directing young adults’ career choices towards specific future careers. As Arur and Sharma [80] suggest, reflecting on the SDGs in a career guidance setting can help clients make connections between their aspirations and the desired futures for themselves, others, and society, enabling them to reframe their narratives accordingly. 

### 6.2. Implication for HE Practices 

These findings can provide insights into the potential benefits of integrating awareness of sustainable development into educational and policy strategies, aiming to promote positive career outcomes and overall well-being. Universities, as well as other educational systems, should always be concerned with what the role of education is today. In the context of higher education and sustainable development, Gough and Scott [81,82] asked whether universities exist to serve the economy or to contribute to the intellectual and also moral betterment of society. Students should be equipped with the necessary skills to face the challenges of society and to build a better society [83,84].

Higher education (HE) could play a leading role in achieving the SDGs. Along with providing academic knowledge and technical skills, several studies [85] have emphasized that universities are also responsible for developing students’ behaviors towards a more sustainable society. They have a duty to provide students with the necessary knowledge, skills, and values, as well as motivation and encouragement, to tackle complex sustainability challenges and contribute to achieving the SDGs [86]. Universities therefore have a responsibility to educate tomorrow’s workforce with the knowledge and skills required to tackle sustainable development challenges [87]. Likewise, university students play an active role in this process, as they are in training, and their abilities need to be developed to implement the SDGs in line with their respective areas of expertise [88]. 

## 7. Study Limitations and Future Perspectives

It is important to acknowledge the limitations of this study. First, the measures utilized to assess the SDGs, courage, proactive career behaviors, and life satisfaction are self-report measures. As is well known, responses to self-report questionnaires could be influenced by potential biases, such as social desirability and interpretation of items. Secondly, this study is cross-sectional. This method does not permit the establishment of causality or the determination of the direction of relationships between study variables. Thirdly, as our sample is exclusively from the South of Italy, the results may not be generalizable. Fourthly, there is a challenge in assessing the accuracy of responses in terms of knowledge of the content of the SDGs. Assessing respondents’ propensity to believe that the SDGs may influence future careers does not automatically provide information on their actual level of knowledge of the goals, let alone whether they will actually choose sustainable career paths.

Nevertheless, the limitations identified suggest several avenues for future research. First, future studies should be conducted using a longitudinal design in which each step in the mediation chain is assessed at consecutive time points. This design would assess whether improvements in the SGDs influence courage and proactive career behaviors and, conversely, whether life satisfaction orientation influences the SDGs or vice versa. Additionally, longitudinal qualitative or quantitative studies could be beneficial to examine the career trajectories of university students to determine whether their awareness of global challenges and their role in addressing them actually influences their career transitions and choices after graduation.

Furthermore, it would be beneficial to examine the relationship between the SDGs and other career outcomes in order to expand current knowledge and inform the design of career guidance interventions. For example, it would be worthwhile to examine the relationship between the SDGs and career indecision or exploration. Finally, future studies should also expand our understanding of the relationship between the SDGs and well-being by examining the potential mediating role of the meaning in life.

## 8. Conclusions

In light of the link between the SDGs and career and well-being outcomes, there is a clear need to focus more on the potential contribution of the SDGs for working with youth within and beyond education systems to build a sustainable future and guide them in this direction. Given the threats and challenges of contemporary society, young people should be aware of how sustainable goals can influence their future education and careers.

This study did not aim to focus on the strategies or initiatives implemented by universities to achieve the 2030 Agenda goals (such as reforms in student education). Instead, it seemed to illuminate the significance of fostering students’ awareness of the processes involved in shaping their future, both in terms of education and careers, as it serves as a catalyst for proactive behaviors and overall well-being.

## Figures and Tables

**Table 1:** Descriptive statistics and correlation between study variables.

	Variable	M	SD	Sk	K	1	2	3	4	5	6
1.	Gender	-	-	-	-	-					
2.	Curriculum	-	-	-	-	0.042	-				
3.	Life satisfaction	4.93	1.34	−0.546	−0.141	0.007	0.027	-			
4.	Proactivity	4.85	0.42	−1.678	−0.898	0.070	0.055	0.573 **	-		
5.	Courage	5.56	0.96	−0.517	−0.257	0.112 *	0.039	0.397 **	0.374 **	-	
6.	SDGs	4.57	0.55	−0.564	1.948	−0.016	−0.001	0.463 **	0.566 **	0.314 **	-

Note: “SDGs” indicates the propensity to consider the SDGs in relation to future education and career paths; M = mean; SD = standard deviation; Sk = skewness; K = kurtosis. Gender (0 = male; 1 = female); university curriculum (0 = science field; 1 = humanities field). * *p* < 0.05; ** *p* < 0.001.

## Data Availability

The data presented in this study are available on reasonable request from the corresponding author. The data are not publicly available due to privacy reasons.
